# Mimosa: Mixture model of co-expression to detect modulators of regulatory interaction

**DOI:** 10.1186/1748-7188-5-4

**Published:** 2010-01-04

**Authors:** Matthew Hansen, Logan Everett, Larry Singh, Sridhar Hannenhalli

**Affiliations:** 1 Department of Genetics, Penn Center for Bioinformatics, University of Pennsylvania, Pennsylvania, USA

## Abstract

**Background:**

Functionally related genes tend to be correlated in their expression patterns across multiple conditions and/or tissue-types. Thus co-expression networks are often used to investigate functional groups of genes. In particular, when one of the genes is a transcription factor (TF), the co-expression-based interaction is interpreted, with caution, as a direct regulatory interaction. However, any particular TF, and more importantly, any particular regulatory interaction, is likely to be active only in a subset of experimental conditions. Moreover, the subset of expression samples where the regulatory interaction holds may be marked by presence or absence of a modifier gene, such as an enzyme that post-translationally modifies the TF. Such subtlety of regulatory interactions is overlooked when one computes an overall expression correlation.

**Results:**

Here we present a novel mixture modeling approach where a TF-Gene pair is presumed to be significantly correlated (with unknown coefficient) in an (unknown) subset of expression samples. The parameters of the model are estimated using a Maximum Likelihood approach. The estimated mixture of expression samples is then mined to identify genes potentially modulating the TF-Gene interaction. We have validated our approach using synthetic data and on four biological cases in cow, yeast, and humans.

**Conclusions:**

While limited in some ways, as discussed, the work represents a novel approach to mine expression data and detect potential modulators of regulatory interactions.

## Background

Eukaryotic gene regulation is carried out, to a significant extent, at the level of transcription. Many functionally related genes, e.g., members of a pathway, involved in the same biological process, or whose products physically interact, tend to have similar expression patterns [1,2]. Indeed, co-expression has been used extensively to infer functional relatedness [3-6]. Various metrics have been proposed to quantify the correlated expression, such as Pearson and Spearman correlation [2], and mutual information [5]. However, these metrics are symmetric and they neither provide the causality relationships nor do they discriminate between indirect relations. For instance, two co-expressed genes may be co-regulated, or one may regulate the other, directly or indirectly.

A critical component of transcription regulation relies on sequence-specific binding of transcription factor (TF) proteins to short DNA sites in the relative vicinity of the target gene [7]. If one of the genes in a pairwise analysis of co-expression is a TF then the causality is generally assumed to be directed from the TF to the other gene. In the absence of such information, an additional post-processing step [5] can be used to infer directionality between the pair of genes with correlated expression. Moreover, a first order conditional independence metric [4] has been proposed to specifically detect direct interactions.

While TFs are the primary engines of transcription, their activity depends on several other proteins such as modifying enzymes and co-factors, which directly or indirectly interact with the TF to facilitate its activity. For instance, the activity of TF CREB depends on a number of post-translational modifications, most notably, Ser133 phosphorylation by Protein Kinase A [8]. Moreover, for many TFs, the TF activity is likely to be restricted to specific cell types and/or experimental conditions. Thus the common practice of using large compendiums of gene expression data to estimate co-expression and thus functional relatedness has two main limitations: (1) it includes irrelevant expression samples which adds noise to the co-expression signal, and (2) it overlooks the contributions of additional modifier genes and thus fails to detect those modifiers which are critical components of gene regulatory networks.

To infer the dependence of TF activity on histone modification enzymes, Steinfeld et al. analyzed the expression of TF-regulons (putative targets of a TF) in yeast samples where specific histone modification enzymes were knocked out [9]. In a different study, Hudson et al. analyzed two sets of expression data in cow, a less-muscular wild-type and another with mutant TF Myostatin [10]. They found that the co-expression of Myostatin with a differentially expressed gene, MYL2, was significantly different between the mutant and the wild-type sets of expression. This differential co-expression led them to detect Myostatin as the causative TF even though the expression of Myostatin gene itself was not different between the mutant and the wild type. In both of the cited examples [9,10], the two sets of expression were well characterized and known a priori. In fact, Hu et al. have proposed a non-parametric test to detect differentially correlated gene-pairs in two sets of expression samples [11]. However, it is not clear how to detect such differentially co-expressed gene pairs when the appropriate partition of the expression samples is not provided and cannot be derived from the description of the experiments. This problem is an important practical challenge for large expression compendiums that cover many diverse experimental conditions. The tremendously growing expression compendium [12], provides a unique opportunity to identify not only co-expressed and functionally related genes, but also to predict putative modifiers of gene regulators.

For a pair of genes for which we have expression data across a set of conditions/samples, we assume there is some partition of the conditions such that the two genes are correlated in one partition and are uncorrelated in the other. Here we propose a novel approach, "Mimosa", that detects the hidden partition of the expression samples into correlated and uncorrelated subsets. If found, such a partition suggests the existence of modifier genes, such as TF modifying enzymes, that should be differentially expressed between the correlated and uncorrelated sample partitions. In other words, genes whose expression vector across samples is correlated with the sample partition vector are putative modifiers. The sample partition is derived from a mixture model of the co-expression data. The free parameters of the mixture model are estimated using a Maximum Likelihood Estimation (MLE) approach. Once the mixture parameters are obtained, we can then compute a weighted partitioning of the samples into the correlated and uncorrelated sets. In a subsequent step, we detect putative modifier genes that are differentially expressed between correlated and uncorrelated samples. Using synthetic data we show that Mimosa can partition expression samples and detect modifier genes with high accuracy. We further present four biological applications, one in bovine samples, two in yeast, and one in human B cells. This work represents a novel approach to mine expression data and detect potential modulators of regulatory interactions.

## Methods

### Mixture modeling of co-expression

**Figure 1:**
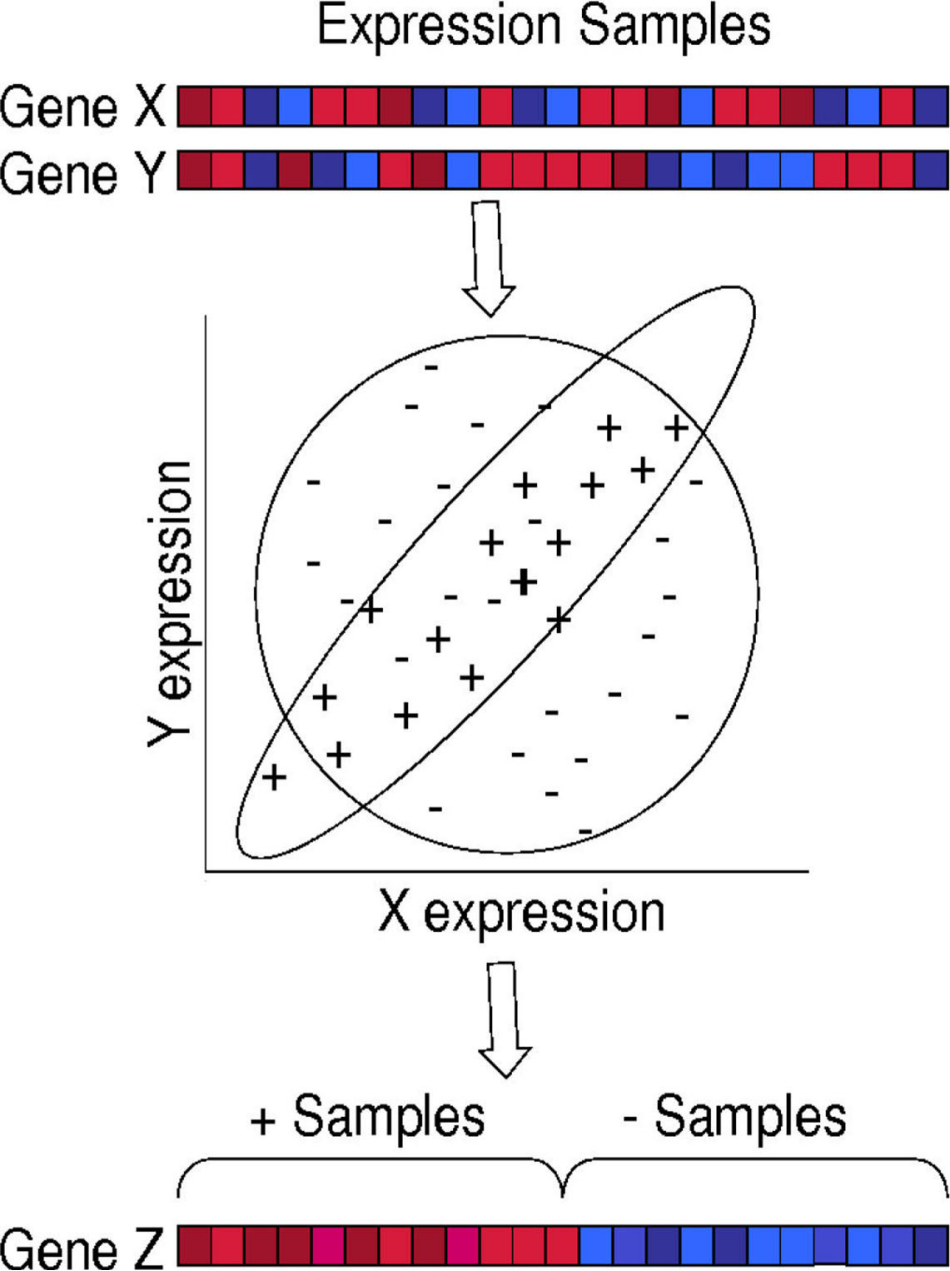
**The figure illustrates the intuition behind Mimosa**. Consider a TF gene *X *and a potential target gene *Y*. The expression values of *X *and *Y *for all expression samples are shown as a heat plot and as a scatter plot. We presume that *X *and *Y *expression are correlated only in an unknown subset of samples (depicted by "+") and not in the remaining samples (denoted by "-"). Mimosa computes the maximum likelihood partition of samples. Then given the sample partition, a third gene *Z *with differential expression between the two partitions may represent a potential modifier. To be precise, we assign a partition probability to each sample as opposed to a binary partition.

Figure 1 illustrates the method. The input data, i.e. the expression profiles, is a matrix *M *[*i*, *k*] where the genes, indexed by *i *= 1, 2, ..., *N*_*g*_, are the rows and the expression samples, indexed by *k *= 1, 2, ..., *N*_*s*_, are the columns of the matrix. *M *[*i*, *k*] represents the expression of gene *i *in expression sample *k*. All rows are normalized to have mean 0 and variance of 1. For each pair of genes *i *and *j*, there are *N*_*s *_data points of expression value pairs, (*M *[*i*, *k*], *M *[*j*, *k*]). For ease of notation, we shall denote the data points as (*x*_*k*_, *y*_*k*_). The observed data set for the gene pair, (*x*_*k*_, *y*_*k*_), is assumed to be a mixture of two different distributions: the group of uncorrelated samples (group "*u*") and the group of correlated samples (group "*c*"), each with its own probability distribution; call these distribution functions *p*_*u*_(*x*, *y*) and *p*_*c*_(*x*, *y*). By definition *p*_*u*_(*x*, *y*) = *p*_*u*_(*x*) *p*_*u*_(*y*), where *p*_*u*_(·) is the normal distribution.

The observed data is viewed as a random sampling from these two groups with mixing fraction *f *defined to be the fraction of data points that belong to the uncorrelated group. The total likelihood of a data point (*x*, *y*) is *p*(*x*, *y*) = *f p*_*u*_(*x*, *y*) + (1 - *f*) *p*_*c*_(*x*, *y*). In the present analysis we assume the uncorrelated distributions to be normal, hence,(1)

We derive the distribution of correlated data, *p*_*c*_(*x*, *y*) by assuming that there is some (*u*, *v*) coordinate system related to the (*x*, *y*) coordinate system by a rotation through an angle *θ*, such that *p*_*c*_(*u*, *v*) = (*u*, *σ*_*u*_) (*v*, *σ*_*v*_). Here, (*x*, *σ*) is the Gaussian distribution with zero mean and variance *σ*^2^. The coordinate transformations from (*x*, *y*) coordinates to (*u*, *v*) coordinates are: *u *= *x *cos *θ*- *y *sin *θ *and *v *= *x *sin *θ *+ *y *cos *θ*. The Jacobian of the transformation is 1, so we have(2)

There are three unknowns, {*θ*, *s*_*u*_, *s*_*v*_}. There are, however, two natural constraints on the form of *p*_*c*_(*x*, *y*); namely, that(3)

Applying these two constraints to eqn. (2), and assuming that *σ*_*u *_≠ *σ*_*v*_, we have(5)

where -1 ≤ *α *≤ 1 is a free parameter of the mixture model that controls the aspect ratio of the correlated distribution. Without loss of generality let *σ*_*v *_>*σ*_*u*_; then in terms of *α *we have *σ*_*u*_^2 ^= (1 - |*α*|) and *σ*_*u*_^2 ^= (1 + |*α*|). Note that *α *< 0 corresponds to positively correlated data (*θ *= *π*/4) and *α *> 0 corresponds to negatively correlated data (*θ *= -*π*/4). For an aspect ratio defined by *r *≡ *σ*_*v*_/*σ*_*u*_> 1, we have |*α*| = (*r*^2^-1)/(*r*^2^+1). In summary, the mixture model has two free parameters, (*f*, *α*), that determine the fraction of uncorrelated points in the observed data and the aspect ratio of the distribution for correlated data.

The log likelihood of the observed data is(6)

We maximize *L *numerically using the quasi Newton-Raphson function optimization routine in the open source Gnu Scientific Library http://www.gnu.org/software/gsl. The resulting parameter estimates are  and .

For each selected gene pair, we compute the probability that each sample belongs to the correlated group. For the *k*^*th *^sample, this is given by(7)

This vector of probabilities is equivalent to a weighted partitioning of the sample set. Modifier genes are selected based on their correlation with vector . We compute this correlation with a t-test based on the expected population number, mean, and variance (see below). When computationally feasible, we use non-parametric correlation measures, such as Kendall's Tau.

### Weighted t-statistic

Given two vectors: (1) the  vector denoting the partition probability for each sample, and (2) expression vector  over all samples for a potential modifier gene, we can, in principle, partition the expression samples into two parts based only on the partition probability, and then compare the expression values in the two parts using a t-statistic or an alternative non-parametric test. However, this approach requires an arbitrary choice of partition probability threshold to partition the sample. We instead used a weighted version of the t-statistic that obviates the need for an arbitrary threshold. The standard t-statistic requires three parameters for each of the two partitions: the two sample-means, the two sample-standard deviations, and the two sample-sizes. We computed all these parameters using a weighted sum. For instance, the sample mean of the correlated partition, *μ*_*c*_, can be estimated as , where  is the weighted number of correlated samples. Similarly, the standard deviation of the correlated partition, *σ*_*c*_, is given by .

### Generating synthetic data

To generate a TF-Gene-Modifier triplet for a given *f *and *α *we performed the following steps. We first create the modifier and TF expression data independantly by random sampling from a normal distribution. For the given *f*, we determine the modifier expression threshold m * such that below this threshold the TF and gene are presumed to be uncorrelated and above this threshold, the TF and the gene are presumed to be correlated. The value of m * is estimated by . We generate the gene expression value as follows. Let *m *be the modifier expression in the *k*^*th *^sample. If m < m*, then the gene's expression value for that sample, *y*_*k*_, is drawn from a normal distribution (the uncorrelated distribution). If *m *≥ m*, then the gene's expression value is drawn from a Gaussian distribution with mean -*αx*_*k *_and variance (1 - *α*^2^), where *x*_*k *_is the expression value of the TF for the *k*^*th *^sample. The latter step follows from the fact that the co-expression distribution for correlated data can be written as *p*_*c*_(*x*, *y*) = *p*_*u*_(*x*)*p*_*c*_(*y|x*) where *p_c_*(*y|x*) is a Gaussian with mean -*αx *and variance (1 - *α*^2^).

## Results and Discussion

### Synthetic Data

Given a pair of genes with a mixed set of correlated and uncorrelated samples, and also a modifier gene whose expression is correlated with the two types of samples, we tested whether our method can detect the correct modifier, which implicitly requires the correct identification of the sample partition. Details of the simulation are provided in §Methods. We generated 1500 non-overlapping TF-Gene-Modifier triplets and for each gene in the triplet we generated the expression data for 300 samples based on an underlying model, parameterized by *f *and *α*. We selected a range of parameters and tested the effect of these parameters on the method accuracy. Intuitively, Mimosa will work best for values of *f *near 1/2 and for values of *α *close to ± 1. Five different values of *f *were chosen that broadly encompass the value of *f *= 0.5. As the sign of *α *does not affect Mimosa's ability to partition the data samples, we chose only positive values of *α*. The three values of *α *chosen were based on their corresponding aspect ratios (see §Methods); namely aspect ratios of 2, 3, and 5. Not surprisingly, the performance of Mimosa deteriorates for aspect ratios below 2, that is, when the correlation is very poor even for the correlated samples (not shown). Each parameter bin contained 100 TF-Gene-Modifier triplets (15 bins × 100 triplets per bin = 1500 triplets, and 3 × 1500 triplets = 4500 total genes). For each of the 1500 TF-Gene pairs, we applied Mimosa to estimate the sample partition and then ranked all 4500 genes based on the weighted t-test p-value of their partitioned expression values (see §Methods). For each 2-dimensional bin (*f *and *α *value), we computed the median rank (out of 4500 candidates) of the correct modifier for the 100 TF-Gene pairs in the bin. We also computed the fraction of the 100 TF-Gene pairs for which the correct modifier had the highest rank.

As shown in Table 1, Mimosa detects the correct sample partition and the correct modifier with high accuracy. Overall, in 64.6% of the cases, the correct modifier is detected at the top rank. When the TF-Gene pair is uncorrelated in 90% of the samples (last column) then it is relatively difficult to detect the modifier. Even then, if the correlation is strong (aspect ratio of 5) then Mimosa can still detect the modifier with very high accuracy. Note that the highest median rank, 215 for the *α *= 0.6 and *f *= 0.9 bin, when represented as a percentile out of 4500 candidates, is only 215/4500 = 4.8%.

**Table 1:** Performance of Mimosa on synthetic data.

*α*(*r*)/*f*	0.1	0.25	0.5	0.75	0.9
0.6 (2)	44, 14%	1, 53%	1, 76%	7, 32%	215, 5%

0.8 (3)	1, 70%	1, 99%	1, 100%	1, 83%	35, 10%

0.923 (5)	1, 99%	1, 100%	1, 100%	1, 99%	4, 30%

Columns represent *f *ranges and rows represent *a *ranges (corresponding aspect ratio is shown in parenthesis; see §Methods). Figures in each cell are based on 100 TF-Gene pairs, and shows (1) the median rank of the correct modifier, and (2) the fraction of 100 cases where the correct modifier was top ranked based on the t-test p-value.

### Application to Bovine data

Hudson et al., have compared expression profiles in two different genetic crosses (denoted *P *and *W*) of cattle at different developmental time points. The *P* type has a mutant form of TF Myostatin which results in dysregulation of TGF-*β *pathway and increased muscle mass [10]. The expression level of Myostatin was not different in these two types. They further identified differentially expressed genes between *P *and *W*, and for each such gene, and for each of the 920 putative regulators, they computed the expression correlation between the gene and the regulator, separately in *P *and in *W *samples. Based on these pair-wise correlations in the two sets of samples, they identified 424 regulator-gene pairs such that the expression correlation between the two was significantly different when using expression data from *P *compared with the expression correlation when using expression data from *W*. This data provides an ideal test bed for our approach.

We tested how well Mimosa partitions the expression samples into *P *and *W *without any prior knowledge. We subjected each of the 424 regulator-gene pairs to the mixture modeling, using the 20 expression profiles (10 for *P *and 10 for *W*) provided in [10]. This resulted in 424 partition probability vectors , each of length 20 (see §Methods). If the mixture modeling is effective, we expect {*q*_1_, ..., *q*_10_} (corresponding to *P*) to be significantly different from {*q*_11_, ..., *q*_20_} (corresponding to *W*), with one being high, and the other being low. We tested this hypothesis using the Wilcoxon test and found that for 109(26%) of the 424 pairs, the p-value ≤ 0.05. Thus the mixture modeling correctly retrieves the hidden sample partition in many cases, even with a small number of expression samples.

### Application to Yeast

**Figure 2:**
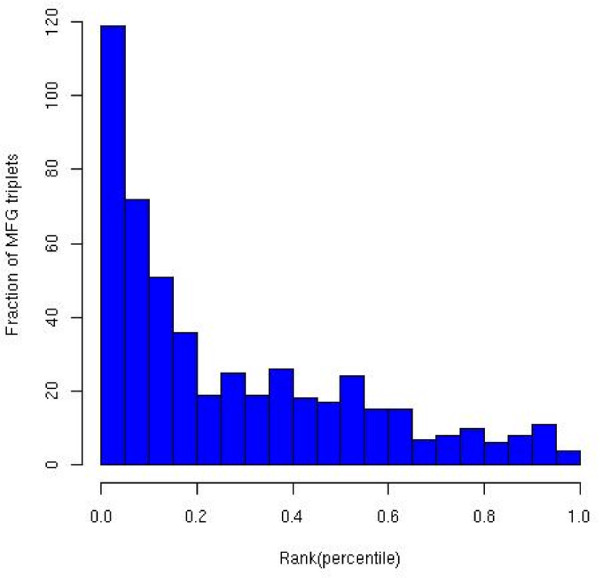
**Distribution of percentile ranks of the correct modifier predicted from among 6000 candidate modifiers, for the 510 experimentally determined TF-Gene-Modifier triplets**. Mimosa ranks the correct modifier among the top 5% in 23% of the cases.

We have previously reported a database - PTM-Switchboard [13], which now contains 510 yeast gene triplets, termed "MFG-triplets", where a transcription factor (F) regulates a gene (G) and this regulation is modulated by post-translational modification of F by a modifying enzyme (M). We tested whether, for the given F-G pair, Mimosa can correctly partition a set of expression samples and detect the modifier M. For the expression data, we used 314 *S. cerevisiae *expression samples previously compiled in [14] from 18 different studies. These experiments included cell cycle and a variety of stress conditions. We applied Mimosa to each F-G pair and then computed the correlation (using Kendall's Tau) of the sample partition probability vector  (see §Methods) and the expression vector of all 6000 yeast genes. We then computed the ranks (in percentile) of the correct modifiers. As shown in Figure 2, we found that Mimosa detects the true modifier among the top 5% in 23% of the cases, a ~5-fold enrichment over random expectation.

To test the large-scale applicability of Mimosa, we extracted all yeast TF-Gene pairs detected in a genome-wide ChIP-chip experiment [15]. To reduce the number of gene-pairs to be tested we performed the following filtering steps. For each pair we computed their expression correlation using Kendall's Tau across the 314 expression samples. We retained the pairs for which the Kendall's Tau Bonferroni-corrected p-value ≤ 0.05. After applying Mimosa, we further filtered this set to retain only the cases where the mixing probability parameter *f *was between 0.45 and 0.55 and the aspect ratio parameter *α *had an absolute value of at least 0.8 (highly correlated). For each of the 6960 TF-Gene pairs thus obtained we computed the corresponding partition probability vector .

**Figure 3:**
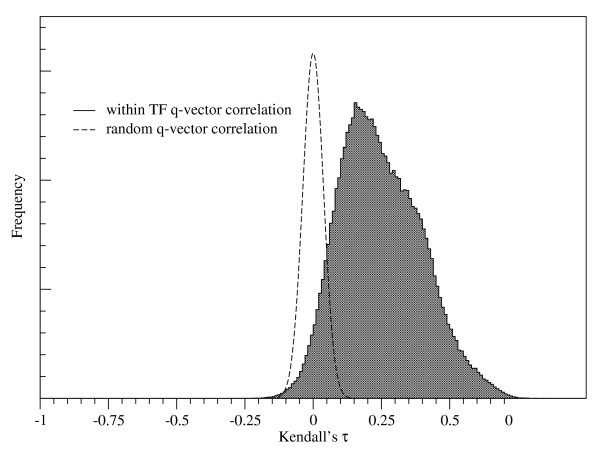
**The distribution of correlations among *q*-vectors with the same TF are shown, and compared to a distribution of correlations for vectors of random numbers**. The data used is taken from yeast TF-Gene pairs; specifically, the 6960 yeast TF-Gene pairs detected by Mimosa (see text).

Each TF has a set of *q*-vectors, one corresponding to every target gene of the TF. Biologically, we expect the partitioning of samples into correlated and uncorrelated to depend mainly on whether or not the TF is active. If this were the case, then there should be a correlation between the set of *q*-vectors for a TF. As shown in Figure 3, the Kendall Tau correlation among *q*-vectors with the same TF does indeed have a distribution that is significantly skewed towards positive values, relative to the correlations between randomly chosen *q*-vectors. This result provides some evidence that the *q*-vector partition found by Mimosa contains biological information.

We then calculated the correlation between every gene's expression vector  and each pair's  vector. "Modifiers" for each pair were deemed to be those genes whose correlation qualified a Bonferroni-corrected, weighted t-statistic p-value threshold of 0.05. We used a weighted t-statistic, as opposed to Kendall's Tau, primarily for computational efficiency. We then performed a functional enrichment analysis on the 1356 putative modifier genes thus obtained using the DAVID tool (david.abcc.ncifcrf.gov). Table 2 shows the enriched (FDR < 5%) molecular functions sorted by the fraction of input genes annotated to have that function. The most abundant molecular function category was "catalytic activity", which is consistent with the role of modifying enzymes. This enrichment holds even when we selected the single most significant modifier for each TF-Gene pair. Further work needs to be done to analyze the biological significance of specific modifiers detected.

**Table 2:** GO molecular functions enriched in the putative modifiers detected for the TF-Gene pairs in *S. cerevisiae* based on ChIP-chip data and 314 expression samples.

Molecular function term	% Coverage	p-value	FDR (%)
catalytic activity	43	1.32E-04	0.22

nucleotide binding	14	1.27E-05	0.02

purine nucleotide binding	13	4.74E-05	0.08

purine ribonucleotide binding	12	1.49E-05	0.02

ribonucleotide binding	12	1.49E-05	0.02

RNA binding	11	1.60E-09	2.71E-06

structural molecule activity	10	4.34E-12	7.36E-09

structural constituentof ribosome	9	4.38E-22	7.43E-19

GTP binding	3	7.43E-06	0.01

guanyl nucleotide binding	3	7.43E-06	0.01

guanyl ribonucleotide binding	3	7.43E-06	0.01

oxidoreductase activity, acting on CH-OH group of donors	3	4.66E-05	0.08

translation regulator activity	3	8.71E-07	1.48E-03

oxidoreductase activity, acting on the CH-OH group of donors, NAD or NADP as acceptor	3	1.10E-04	0.19

translation factor activity, nucleic acid	3	2.14E-07	3.62E-04

GTPase activity	2	2.39E-03	3.98

rRNA binding	2	8.85E-11	1.50E-07

snoRNA binding	2	8.58E-09	1.45E-05

ligase activity, forming aminoacyl-tRNA and related compounds	2	1.33E-06	2.25E-03

ligase activity, forming carbon- oxygen bonds	2	1.33E-06	2.25E-03

aminoacyl-tRNA ligase activity	2	1.33E-06	2.25E-03

RNA helicase activity	2	4.29E-05	0.07

ATP-dependent RNA Helicase activity	2	9.12E-07	1.54E-03

RNA-dependent ATPase activity	2	9.12E-07	1.54E-03

translation initiation factor activity	1	4.61E-04	0.78

### Application to STAT1

Transcription factor STAT1 plays a critical role in B cell function and B cell cancers [16]. STAT1 activity is known to be controlled via a variety of post-translational modifications [17-20]. We attempted to detect potential upstream modulators of STAT1 in B cells using Mimosa. We obtained a set of genes from [21] reported to be STAT1 targets and manually mapped these to 50 transcripts. We also obtained a compendium of 336 expression samples in human B cells from [6], which includes samples from human blood, cancers, and cell lines based on the HG-U95Av2 Affymetrix arrays. We then applied Mimosa to all pairs consisting of a STAT1 probe and a probe corresponding to one of the targets. Applying the criteria of 0.3 ≤ *f *≤ 0.7 and |*α*| ≥ 0.8, we obtained 10 targets whose expressions were correlated with that of STAT1 in a subset of samples. We then detected 34 genes whose expression was correlated with partition vector  (see Methods) with a p-value ≈ 0.

The 34 detected include a number of modifying enzymes such as kinases and phosphotases, as well as transcription factors and co-factors, and membrane receptors. A number of the genes are involved in or peripherally related to IFN-gamma signaling, which is the major activator of STAT1 [22], as well as TGF-beta and NF-kappaB signaling, both of which are important in B cell apoptosis/survival. Several of the the detected genes, namely GRK5 and UBE21, have known roles in JAK-STAT signaling. It is possible that these detected genes may play a mechanistic role in the cross-talk between pathways affecting STAT1 activity. However, we cannot rule out the possibility that some of these genes actually operate downstream of or in parallel to STAT1, in which case their correlation with the partition vector  is due to some shared and undetected upstream modulator. We have summarized these findings for 24 of the 34 genes in Table 3. We could not find any plausible link with STAT1 for the other 10 genes.

**Table 3:** Potential modulators of STAT1 activity detected by Mimosa using the known STAT1 targets and gene expression data from normal B cell and B cell cancers.

Gene Name	Evidence
Refseq Id	[Pubmed Id for the references are provided in square brackets]
	**Modifying Enzymes**

*GRK5* NM005308	A Ser/Ther protein kinase that functions upstream of the JAK-STAT signal transduction pathway according to the KEGG pathway database http://www.genome.jp/kegg.

*UBE21* NM194261	An E2 SUMO-conjugating enzyme implicated in SUMOylation of STAT1 in conjunction with PIAS1 [12855578, 12764129].

*DUSP1* NM004417	A dual specificity protein phosphatase. STAT1 is known to be primarily regulated by reversible tyrosine phosphorylation. DUSP1 has been shown to function in a JAK2-dependent manner [14551204] and the members of the JAK family are the canonical regulators of STATs, thus suggesting DUSP1 as a potential upstream modulator of STAT1.

*SIK1* NM173354	A Ser/Thr kinase that negatively regulates the TGF-*β *pathway [18725536]. IFN-*γ *signaling is mediated via STAT1, while TGF-*β *and IFN-*γ *pathways are known to be directly antagonistic to each other [17116388], thus suggesting a role for SIK1 modulation of STAT1 in pathway cross-talk.

*INPP1* NM002194	A phosphatase functioning upstream of major kinases such as AKT/PKB (KEGG pathway), which are known to mediate apoptotic signaling in B cells [17928528].

	**Receptors**

*CD69* NM001781	An early activation antigen functioning downstream of IFN-*γ *[12718936], and STAT1 activation is known to be interferon-responsive.

*LGALS8* NM201543	Modulates cellular growth through up-regulation of p21 [15753078], which in turn is regulated by the STAT1 homolog STAT5A [12393707].

*SELL* NM000655	Belongs to a family of adhesion/homing receptors which play important roles in leukocyte-endothelial cell interaction [12370391], while STAT1 also plays a crucial role in leukocyte-infiltration into the liver in T cell hepatitis [15246962].

	**Transcription factors and co-factors**

*DIP* NM198057	Glucocorticoid-induced leucine zipper (GILZ) interacts with NF-kappaB [17169985] which is known to play a key role in B cell function.

*IRF7* NM004031	An interferon regulatory factor 7, belonging to the same TF family as two known STAT1 co-factors, IRF-1 and IRF8 [18929502].

*POLR2J* NM006234	Co-induced with STAT3 by HIV-1 gp120 [12089333].

*POLR2J2* NM032959	Related to POLR2J.

*ZNHIT3* NM004773	A zinc finger transcription factor known to be a HNF-4*α *co-activator [11916906]. However, we did not find a potential link with STAT1.

	**Other Immune Related Genes**

*ADRM1* NM007002	A proteasomal ubiquitin receptor whose expression has been shown to be induced by IFN-*γ *[8033103]. STAT1 activity is known to be modulated by ubiquitin-dependent protein degradation [18378670].

*PSMD9* NM002813	A 26S proteasome non-ATPase regulatory subunit involved in the processing of class I MHC peptides [8811196].

*IFITM-1,2,3* NM003641 NM006435 NM021034	Interferon-induced transmembrane proteins. These may be involved in STAT1 modulation, or they may be downstream of a pathway, most likely IFN-*γ*, which modulates STAT1 activity.

*HLA-A,C,E,F,G,L* NM002116 NM002117 NM005516 NM018950 NM002127 NM001004349	MHC class I genes. The function of this class of genes is well-characterized as cell-surface antigen presenters, and it is difficult to imagine how these genes might function upstream of STAT1. A more likely explanation is that they are activated downstream of, or in parallel to, STAT1 by another gene which also functions as a STAT1 modulator or co-factor. It is particularly striking that all of these genes belong to MHC class I, and none in MHC class II, which are known to be regulated by STAT1 [18929502].

## Conclusions

For a pair of co-expressed genes (*X *and *Y*), we have presented a mixture modeling approach to partition the expression samples in order to detect the specific subset of samples where *X *and *Y *expressions are strongly correlated. In some cases, such a partition may help detect other genes likely to modulate the expression correlation between *X *and *Y*. Such a potential modulator is characterized by having differential expression in the two sample partitions. A few previous investigations closely relate to our work. In [10] and in [11], given two sets of expression samples, the authors explicitly search for gene-pairs whose expression correlations are significantly different in the two sets of samples. A different approach, termed Liquid Association, explicitly tries to detect gene triplets (*X*, *Y*, *Z*) where the change in correlation between *X *and *Y *varies with the changes in the value of *Z *[23]. This approach implicitly partitions the expression samples based on the modulator gene expression. In contrast, our approach partitions the expression samples without any knowledge of the modulator gene and proceeds with the search for modulator genes in a subsequent step.

**Figure 4:**
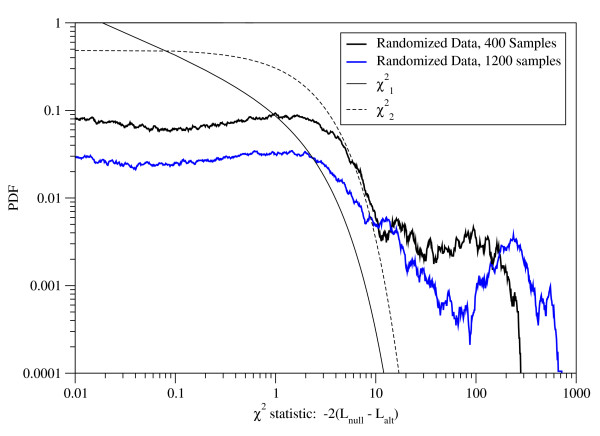
**The figure shows (1) The distribution of Log-Likelihood ratios for randomly generated (normal, i.i.d.) expression data for 400, and 1200 samples, permuted 20,000 times, (2) *χ*^2 ^distributions with 1 and 2 degrees of freedom**. The "null" distribution is defined by *f *= 0, implying an absence of a mixture.

In a genome-wide application, such as in the yeast application presented above, in principle, one can apply a Log-Likelihood Ratio (LLR) test, where the likelihood of the mixture model with a free *f *and *α *parameters is compared with the likelihood of a model where *f *= 0 and only *α *is free. The log of the ratio of the two likelihoods can be used to assess significance of the partition based on a *χ*^2 ^distribution. While it is appealing to use the LLR test to assess the significance of the mixture model, we found that our empirical distribution does not follow a *χ*^2 ^distribution (Figure 4). Our next thought was to use an empirically derived p-value for the mixture likelihood by randomly permuting the expression data. However, the empirical distributions of the likelihood itself varied significantly among different gene-pairs and thus we could not use a global distribution. Unfortunately, the number of permutations desired for an adequately resolved p-value is computationally infeasible if done for each gene-pair separately. Thus, as a practical compromise, in the genome-wide yeast application, we chose to only consider gene-pairs with a Bonferroni-corrected global Kendall's Tau correlation p-value ≤ 0.05.

We face a similar challenge in the second phase of the approach, where, given the mixture model and the sample partition probability vector , we search for modulator genes based on the correlation of their expression vectors with . For a large number of trials (number of candidate modulators), a non-parametric test of correlation, such as Kendall's Tau, becomes infeasible. Thus, as another practical compromise, we devised the weighted t-test, which works well for the synthetic data. For the small-scale yeast application on specific (*X*, *Y*, *Z*)-triplets, we used Kendall's Tau but for the large-scale application we used weighted t-statistic. A more detailed study needs to be done to carefully assess the effect of these practical choices on the method's accuracy and efficacy.

Our mixture modeling may be most effective in cases such as the one described in [10], where the sample partition is clearly characterized by a single (unknown) mutant gene. In most practical situations, based on publicly available compendiums of expression data, this may not be the case. Regulatory relationships in eukaryotes have multiple determinants and it is possible that even if the method does detect the "correct" partition, it may be difficult to evaluate the biological significance of the sample partition based on the differential expression of a single modulator gene.

In summary, our work contributes a novel approach to the problem of partitioning expression samples and detecting potential modulators of expression correlation between a pair of genes. While this approach is likely to be effective in specific cases, as discussed above, statistical and computational challenges remain to be resolved and further work needs to be done to harness the approach in a large-scale application.

## Competing interests

The authors declare that they have no competing interests.

## Authors' contributions

SH, LE, and MH conceived the project. MH developed the algorithm and implemented it. LS helped with microarray data processing and general statistical issues. LE helped with STAT1 analysis. SH and MH wrote the manuscript.
